# CXCL10 could drive longer duration of mechanical ventilation during COVID-19 ARDS

**DOI:** 10.1186/s13054-020-03328-0

**Published:** 2020-11-02

**Authors:** Mathieu Blot, Marine Jacquier, Ludwig-Serge Aho Glele, Guillaume Beltramo, Maxime Nguyen, Philippe Bonniaud, Sebastien Prin, Pascal Andreu, Belaid Bouhemad, Jean-Baptiste Bour, Christine Binquet, Lionel Piroth, Jean-Paul Pais de Barros, David Masson, Jean-Pierre Quenot, Pierre-Emmanuel Charles, Pneumochondrie study group, François Aptel, Auguste Dargent, Marjolaine Georges, Marie Labruyère, Laurent Lagrost, Audrey Large, Serge Monier, Jean-Baptiste Roudaut, Charles Thomas

**Affiliations:** 1 Infectious Diseases Department, Dijon Bourgogne University Hospital, Dijon, France; 2 INSERM, LNC UMR 1231, FCS Bourgogne-Franche Comté, LipSTIC LabEx, F-21000 Dijon, France; 3 Department of Pneumology, Dijon Bourgogne University Hospital, Dijon, France; 4 Anesthesiology and Critical Care Department, Dijon Bourgogne University Hospital, Dijon, France; 5 Laboratory of Virology, Dijon Bourgogne University Hospital, Dijon, France; 6 INSERM, CIC1432, Clinical Epidemiology unit; Dijon Bourgogne University Hospital, Clinical Investigation Center, Clinical Epidemiology/Clinical trials unit, Dijon, France; 7 Lipidomic Analytic Unit, University Bourgogne Franche-Comté, Bâtiment B3, Bvd. Maréchal de Lattre de Tassigny, 21000 Dijon, France; 8 Laboratory of Clinical Chemistry, Dijon Bourgogne University Hospital, Dijon, France; 9 Department of Intensive Care, Dijon Bourgogne University Hospital, Dijon, France; 10 Epidemiology and Hospital Hygiene Department, Dijon Bourgogne University Hospital, Dijon, France

**Keywords:** Acute respiratory distress syndrome, COVID-19, SARS-CoV-2, Mechanical ventilation, Immune response, Bronchoalveolar lavage, CXCL10, Mitochondrial DNA, Biomarker

## Abstract

**Background:**

COVID-19-related ARDS has unique features when compared with ARDS from other origins, suggesting a distinctive inflammatory pathogenesis. Data regarding the host response within the lung are sparse. The objective is to compare alveolar and systemic inflammation response patterns, mitochondrial alarmin release, and outcomes according to ARDS etiology (i.e., COVID-19 vs. non-COVID-19).

**Methods:**

Bronchoalveolar lavage fluid and plasma were obtained from 7 control, 7 non-COVID-19 ARDS, and 14 COVID-19 ARDS patients. Clinical data, plasma, and epithelial lining fluid (ELF) concentrations of 45 inflammatory mediators and cell-free mitochondrial DNA were measured and compared.

**Results:**

COVID-19 ARDS patients required mechanical ventilation (MV) for significantly longer, even after adjustment for potential confounders. There was a trend toward higher concentrations of plasma CCL5, CXCL2, CXCL10, CD40 ligand, IL-10, and GM-CSF, and ELF concentrations of CXCL1, CXCL10, granzyme B, TRAIL, and EGF in the COVID-19 ARDS group compared with the non-COVID-19 ARDS group. Plasma and ELF CXCL10 concentrations were independently associated with the number of ventilator-free days, without correlation between ELF CXCL-10 and viral load. Mitochondrial DNA plasma and ELF concentrations were elevated in all ARDS patients, with no differences between the two groups. ELF concentrations of mitochondrial DNA were correlated with alveolar cell counts, as well as IL-8 and IL-1β concentrations.

**Conclusion:**

CXCL10 could be one key mediator involved in the dysregulated immune response. It should be evaluated as a candidate biomarker that may predict the duration of MV in COVID-19 ARDS patients. Targeting the CXCL10-CXCR3 axis could also be considered as a new therapeutic approach.

**Trial registration:**

ClinicalTrials.gov, NCT03955887

## Background

Since December 2019, the world is experiencing an outbreak of coronavirus disease 2019 (COVID-19) caused by severe acute respiratory syndrome coronavirus 2 (SARS-CoV-2). Clinical, radiological, and biological differences have been found between COVID-19-related acute respiratory distress syndrome (ARDS) and ARDS from another origin. Indeed, the sudden clinical deterioration observed 1 week after symptom onset, together with deep hypoxemia contrasting with “normal” (> 40 mL/cmH_2_O) lung compliance, and the higher incidence of thromboembolic events suggest that SARS-CoV-2 is driven by a unique pathogenesis resulting in an atypical form of ARDS [1, 2]. Notably, mechanical ventilation appears to be required for twice as long in COVID-19 than in non-COVID-19 ARDS patients [3, 4].

It has been established that the virus invades type 2 alveolar cells and ciliated epithelial cells that express ACE2 [5]. Subsequently, as they die, infected cells release virus particles and intracellular components including molecules likely to act as alarmins (i.e., danger signals), as reflected by rising lactate dehydrogenase (LDH) levels in the plasma [6]. The ensuing recruitment and activation of immune cells lead to lung damage [7]. For now, several studies have established that the hyperinflammatory response (namely the cytokine storm) induced by SARS-CoV-2 is associated with disease severity and could contribute to the development of ARDS [8, 9]. In addition, since most patients need to undergo mechanical ventilation in this context, ventilator-induced lung injury (VILI) could exacerbate tissue damage as well as local and systemic inflammation, thus acting as a “second hit.” Our team has previously shown that mitochondrial alarmins (i.e., mitochondrial DNA) are released by human epithelial cells submitted to cyclic stretch, and these alarmins are also recovered from bronchoalveolar lavage (BAL) fluid obtained from either ventilated rabbits or ARDS patients. By promoting chemotaxis and activation of polymorphonuclear neutrophils (PMNs), mitochondrial alarmins are thought to represent proximal endogenous mediators of VILI and ARDS when they are released by injured alveolar cells [10, 11].

The aim of our study was to compare cytokine response patterns, in both alveolar and systemic compartments, between COVID-19-related ARDS and non-COVID-19-related ARDS (i.e., ARDS complicating pneumonia from another origin). In addition, we sought to establish the extent to which the immune signature could be associated with clinical evolution according to ARDS etiology.

## Methods

### Study design and patient selection

This is an ancillary study of the ongoing prospective PNEUMOCHONDRIE study (ClinicalTrials.gov NCT03955887) which started in June 2019 in three intensive care units and the department of pneumology at the University Hospital of Dijon (France). Patients were eligible if a BAL was considered necessary by the attending physician and if they had (1) pneumonia (≥ 2 acute signs including cough, purulent sputum, dyspnea, chest pain, temperature < 35 °C or ≥ 38.5 °C, and novel radiological pulmonary infiltrate); (2) ARDS (according to the Berlin Definition) [12]; (3) requiring mechanical ventilation (MV); and (4) BAL was performed within 72 h of the start of MV. COVID-19 ARDS patients were eligible if they tested positive for SARS-CoV-2 by reverse transcriptase polymerase chain reaction (RT-PCR) for at least one respiratory sample. The control population consisted of outpatients without fever during the last 15 days, not under mechanical ventilation, and undergoing a BAL for a condition not related to acute infection (evaluation of a pulmonary nodule, chronic interstitial syndrome, or unexplained chronic pulmonary sign). Seven control patients and 7 bacterial ARDS patients were prospectively included between June 11, 2019, and January 20, 2020 (5 weeks before the pandemic COVID-19 started in Burgundy, France). Fourteen COVID-19 ARDS patients were included between March 30 and April 09, 2020. Oral consent was obtained from the patients or their legal representatives. Approval was obtained from the ethics committee (*Comité de Protection des Personnes Sud-Est III*; 2018-035 B), and an amendment was obtained to include supplementary patients with COVID-19-related ARDS.

### Variables of interest, clinical outcomes, and data collection

Demographic data, comorbidities, clinical and biological parameters, and severity scores (Sequential Organ Failure Assessment (SOFA) [13] and the new simplified acute physiology score (SAPS II) [14]) were calculated on the first day of ARDS. Septic shock was defined as persistent hypotension requiring vasopressors and a serum lactate level > 2 mmol/L despite adequate volume resuscitation. Clinical outcomes were recorded up to 30 days after the start of ARDS: 30-day mortality; number of hospital-, ICU-, and ventilator-free days; and hospital-acquired complications (ventilatory-acquired pneumonia (VAP), thrombo-embolic disease). The “ventilator-free days” outcome was defined as the number of days alive from day 1 of ARDS to day 30 during which the patient was breathing without MV. Dedicated clinical research assistants collected all data using a standardized electronic case report form. Automatic checks were generated for missing or incoherent data. All abnormal data were controlled by a physician-scientist.

The concentration of inflammatory cytokines and mitochondrial alarmins (cell-free mitochondrial DNA concentrations) was measured within both the systemic and alveolar compartments.

### Sample collection and analysis

Bronchoalveolar lavage was performed by fiberoptic bronchoscopy as part of standard care with sterile saline at 37 °C. The ten first milliliters of aspirated fluid, reflecting a bronchial sample, was not considered for the study. Ten milliliters of BAL fluid (BALF) was dedicated to the study, transported at 4 °C, and used within 2 h of collection. Fifty microliters of whole BALF was homogenized with 200 μL of Thrombo-Plus (Sarstedt) before cell count by light microscopy. BAL was filtered through a 70-μm cell strainer (Fisher) and centrifuged at 500×*g* for 10 min at 4 °C to remove mucus and cells. The supernatant was then centrifuged again at 3200×*g* for 5 min at 4 °C to remove the remaining debris and stored at − 80 °C until use. In addition, three additional blood tubes (EDTA) were collected just before the BAL procedure (with a maximum delay of 3 h), as part of standard care, and then centrifuged at 2000×*g* for 10 min at 4 °C and stored at − 80 °C until use.

### Measurement of cytokines

Forty-five analytes were quantified in the plasma and BALF using Human XL Cytokine Magnetic 45-plex Luminex® assay (R&D Systems, USA) according to the manufacturer’s instructions: C-C motif chemokine ligand (CCL)2, CCL3, CCL4, CCL5, CCL11, CCL19, CCL20, CD40 ligand, fractalkine, C-X-C motif chemokine ligand (CXCL) 1, CXCL2, CXCL10, epidermal growth factor (EGF), fibroblast growth factor (FGF), FMS-like tyrosine kinase 3 ligand (FLT3L), granulocyte colony-stimulating factor (G-CSF), granulocyte-macrophage colony-stimulating factor (GM-CSF), granzyme B, interferon (IFN)-alpha, IFN-beta, IFN-gamma, interleukin (IL)-1α, IL-1β, IL-1RA, IL-2, IL-3, IL-4, IL-5, IL-6, IL-7, IL-8, IL-10, IL-12, IL-13, IL-15, IL-17A, IL-17E, IL-33, programmed death-ligand 1 (PDL1), platelet-derived growth factor (PDGF)-AA, PDGF-AB/BB, transforming growth factor (TGF)-α, tumor necrosis factor (TNF)-α, TNF-related apoptosis inducing ligand (TRAIL), and vascular endothelial growth factor (VEGF).

### Cell-free mitochondrial DNA

For mitochondrial DNA isolation, collected conditioned media from the plasma and BALF were first centrifuged at 3200×*g* for 5 min to remove cell debris, followed by DNA extraction using Qiagen DNEasy kit (Qiagen, Valencia, CA, USA). Quantitative PCR was performed on a StepOnePlus™ Real-Time PCR (Applied Biosystem), using SYBR green reagent (PowerUp®, Thermo) with one-tenth and one-fiftieth dilutions of the final product of plasma and BALF respectively, using mitochondrial-specific PCR primers for cytochrome C oxidase subunit III (COXIII) and NADH dehydrogenase subunit I [15], in reference to a standard curve of human mitochondrial DNA to quantify the amount of mitochondrial DNA, amplified as previously reported [10, 11, 15].

### SARS-CoV-2 quantification in BALF

RNA extraction was performed on a NucliSENS® easyMAG® platform (Biomerieux, Marcy-l’Étoile, France), from 200 μL of cell-free BALF, and according to the manufacturer’s instructions. Two target genes were amplified and tested simultaneously, namely the RNA-dependent RNA polymerase (RDRP) IP2 and IP4 regions [16]. Amplification was performed using QuantStudio 5 rtPCR Systems (Thermo Fisher Scientific, Waltham, MA, USA). Quantification of the number of RNA copies was done according to a scale ranging from 10^3^ to 10^6^ copies/mL. All positive BALF samples were quantified and expressed as the number of RNA copies/μL.

### Epithelial lining fluid

To correct for dilution of BALF, the ELF concentration of all analytes or SARS-CoV-2 was calculated by multiplying the BALF concentration with a dilution factor and using urea, according to the formula [analyte]_ELF_ = [analyte]_BALF_ × [urea]_plasma_/[urea]_BALF_, as described by Rennard et al. [17]. Determination of the urea concentration in BALF and plasma was performed using the liquid chromatography tandem mass spectrometry (LC-MSMS) method, adapted from Han et al. [18] with modifications. Plasma (10 μL) or BALF (20 μL) were extracted with 1 mL of cold ethanol containing 1000 ng or 50 ng of 13C and 15N2 urea used as internal standard, respectively. High-performance liquid chromatography (HPLC) was performed on an Agilent 1260 LC system coupled to a 6460 QqQ mass spectrometer. The analysis was conducted in a positive selected reaction monitoring mode. Calibration curves using authentic urea standards (Santa Cruz, Dallas, USA) dissolved in water were prepared. Linear regression was used for calculations.

### Statistical analysis

Collected data were described according to the COVID-19 status of the ARDS (i.e., non-COVID-19 or COVID-19) and for the control group. Continuous variables were expressed as median and inter-quartile ranges (IQR) and categorical variables as number and percentages. The univariate analysis consisted of comparisons between variables, according to the COVID-19 status, performed using the chi-square test (Fisher’s exact test if conditions were not met) for percentages and Wilcoxon Mann-Whitney test for medians and IQRs. Subsequently, the association between COVID-19 s non-COVID-19 ARDS and the number of ventilator-free days was assessed using two multivariate median regression models including some other clinically relevant explicative variables: (i) age, septic shock (yes/no) and PaO_2_/FiO_2_ (model 1) and (ii) age and baseline SOFA score (model 2). Then, cytokines were presented by boxplots to visualize potential associations between cytokines and COVID-19 status (those for which differences were observed according to the COVID-19 status or according to physio-pathological considerations). Finally, multivariate median regression models were built to test the association with the most pertinent clinical outcome associated with COVID-19 status in univariate analysis comparison, including the five most relevant covariates (i.e., COVID-19 status and the cytokines that differed the most between COVID-19 and non-COVID-19 ARDS patient, either clinically or with a *p* value below 0.3) and treatments that differ between both groups. In order to avoid overfitting, the choice of variables was not only made on the *p* value but also considering statistical (i.e., collinearity) and physio-pathological considerations. Model variability explicative power was quantified using the *R*^2^ coefficient. Then, mitochondrial DNA levels were compared between controls, non-COVID-19, and COVID-19 ARDS patients using a Kruskal-Wallis analysis of variance test. To account for multiple comparisons, the *p* value was adjusted for a false discovery rate (FDR) using the Benjamini and Hochberg method. Correlations were sought using the Spearman test between the concentration of cytokines, mitochondrial DNA, and outcome, and also ELF concentrations of SARS-CoV-2, and depicted with a heatmap representation and scatter plot. All tests were two-tailed. A *p* value lower than 0.05 was considered statistically significant. All analyses were performed using the Stata (13.1, Stata Corporation, College Station, TX, USA) or Prism software (GraphPad Prism®, version 8.0, San Diego, CA, USA).

## Results

### Characteristics of the study population

Twenty-eight patients were enrolled in this study (7 in the control group, 7 in the non-COVID-19 ARDS group between June 2019 and January 2020, and 14 in the COVID-19 ARDS group between March and April 2020). The control patients were outpatients suffered from sarcoidosis (*n* = 2), pulmonary Langerhans histiocytosis (*n* = 1), pulmonary carcinoma (*n* = 1), scleromyositis (*n* = 1), focal bronchiectasis (*n* = 1), or unknown cause (*n* = 1), and none had any evidence of current infection or acute disease. Bacterial pneumonia was proven in five patients from the non-COVID-19 ARDS group (*Pseudomonas aeruginosa* (*n* = 1), *Legionella pneumophila* (*n* = 3), *Mycoplasma pneumonia* (*n* = 1)), while co-infection occurred in only one COVID-19 ARDS patient (*Staphylococcus aureus*). While demographic and comorbidity data were not statistically different, baseline severity according to the SOFA score in particular was found to be lower in COVID-19 ARDS patients (*p* = 0.045), with a trend toward less frequent septic shock (*p* = 0.120) and a marginally lower baseline arterial lactate levels (*p* = 0.079) at the onset of ARDS (Table 1). According to the Berlin criteria, ARDS severity was marginally significantly different between non-COVID-19 and COVID-19 patients, with a median baseline PaO_2_:FiO_2_ of as low as 68.5 (IQR = 60.9–90.7) and 88.4 (IQR = 79.2–116.6) mmHg, respectively (*p* = 0.067). Moreover, we found no difference in terms of ventilator settings or lung mechanics. Procalcitonin tended to be only slightly higher in COVID-19 ARDS patients than in the other ARDS patients (0.3 [0.2–0.5] vs. 21.6 [3.8–38.7] μg/L, respectively; *p* = 0.167) (Table 1).

**Table 1:** Patient characteristics and outcomes

	Study groups			***p*** value*
	Control group, ***n*** = 7	Non-COVID-19 ARDS, ***n*** = 7	COVID-19 ARDS, ***n*** = 14	
Age (years), median [IQR]	50 [32–54]	54 [43–64]	67 [63–70]	0.085
Male sex, *n* (%)	5 (71)	6 (85)	11 (78)	0.712
BMI, median [IQR]	26.6 [25.7–32.6]	30.1 [27.1–32.2]	28.2 [25.9–30.5]	0.576
**Medical history**				
Cardiovascular disease, *n* (%)	0	1 (14)	1 (7)	1.000
Pulmonary disease, *n* (%)	1 (14)	1 (14)	4 (29)	0.624
Cerebrovascular disease, *n* (%)	1 (14)	1 (14)	1 (7)	1.000
Malignancy, *n* (%)	0	0	2 (14)	0.533
Diabetes mellitus, *n* (%)	1 (14)	0	3 (21)	0.521
Charlson score, *n* (%)	0 [0–0.5]	0 [0–1.5]	0 [0–1.8]	0.935
Chronic alcohol consumption, *n* (%)	0	2 (29)	0	0.100
Tobacco use, *n* (%)	5 (71)	3 (43)	6 (43)	1.000
**Baseline characteristics**				
Septic shock, *n* (%)		4 (57)	2 (14)	0.120
SAPS II score, median [IQR]		33 [21.0–44.5]	26.5 [21–32]	0.349
SOFA score, median [IQR]		6 [5.5–11]	4 [4–5]	0.045
Tidal volume (mL/kg of predicted body weight), median [IQR]		6.2 [5.8–6.7]	5.7 [5.5–6.1]	0.360
PEEP (cm of water), median [IQR]		10 [10–12]	12 [10–13.5]	0.262
Plateau pressure (cm of water), median [IQR]		24 [22–25.5]	22 [20–24]	0.162
Driving pressure (cm of water), median [IQR]		12 [10.5–17.5]	11.5 [8.3–12]	0.162
Respiratory system compliance (mL/cm of water), median [IQR]		35.3 [29.1–43.3]	35.7 [31.6–50]	0.576
PaO_2_:FiO_2_ (mmHg), median [IQR]		68.5 [60.9–90.7]	88.4 [79.2–116.6]	0.067
Arterial pH, median [IQR]		7.29 [7.29–7.47]	7.46 [7.37–7.49]	0.245
PaCO_2_ (mmHg), median [IQR]		39.5 [38.1–43.7]	35.1 [33.3–40.8]	0.287
Lactate level (mmol/L), median [IQR]		1.8 [1.6–3.3]	1.4 [1.1–1.7]	0.079
C-reactive protein (mg/L), median [IQR]	3 [3–5]	274 [97–347]	155 [118–224]	0.550
Procalcitonin (μg/L), median [IQR]		21.6 [3.8–38.7]	0.3 [0.2–0.5]	0.167
ASAT (IU/L), median [IQR]		47 [43–157]	52 [34–100]	0.799
Serum creatinine (μmol/L), median [IQR]		73 [61–137]	65 [54–81]	0.247
Neutrophils (/mm^3^), median [IQR]	4330 [3738–5260]	10,900 [7705–18,120]	5960 [3960–10,515]	0.094
Lymphocytes (/mm^3^), median [IQR]	2350 [1595–5010]	900 [255–1270]	825 [648–1023]	0.913
Monocytes (/mm^3^), median [IQR]	525 [440–760]	410 [275–795]	420 [220–633]	0.455
**Bronchoalveolar lavage fluid**				
Alveolar cells (/mm^3^), median [IQR]	361 [343–889]	2430 [1072–7740]	1426 [879–3029]	0.255
**Treatments**				
Antibiotic multitherapy, *n* (%)		6 (86)	3 (21)	0.016
Hydrocortisone, *n* (%)		4 (57)	5 (36)	0.397
Hydroxychloroquine, *n* (%)		0	4 (29)	0.255
Remdesivir, *n* (%)		0	1 (7)	1.000
High-flow nasal oxygen, *n* (%)		5 (71)	5 (36)	0.183
Non-invasive mechanical ventilation, *n* (%)		3 (43)	1 (7)	0.088
Prone positioning, *n* (%)		6 (86)	8 (57)	0.337
Extracorporeal membrane oxygenation, *n* (%)		2 (29)	0	0.100
Extrarenal purification, *n* (%)		2 (29)	0	0.100
Vasopressors, *n* (%)		5 (71)	9 (64)	1.000
**Outcomes at 30 days**				
Ventilatory acquired pneumonia (VAP), *n* (%)		0	10 (71)	0.004
Antibiotic-free days, median [IQR]		14 [5.5–17]	13 [5.5–17]	0.911
Thrombo-embolic disease, *n* (%)		1 (14)	6 (43)	0.337
Intensive care unit-free days, median [IQR]		10 [0–15.5]	0 [0–12.3]	0.493
Ventilator-free days, median [IQR]		18 [17–21]	8 [0–15]	0.034
Hospital-free days, median [IQR]		0 [0–9.5]	0 [0–3]	0.450
30-day mortality, *n* (%)		1 (14)	3 (21)	1.000

*Comparison between patients with non-COVID-19 and COVID-19 ARDS

### Outcomes

The 30-day mortality rate reached 14% in the non-COVID-19 group and 21% in the COVID-19 group (Table 1). However, the number of ventilator-free days was significantly lower in COVID-19 patients, compared to non-COVID-19 patients (8 [0–15] vs. 18 [17–21]; *p* = 0.034), along with a higher rate of ventilator-acquired pneumonia (10 (71%) vs. 0 (0%); *p* = 0.004). COVID-19 etiology remained associated with a fewer ventilator-free days within the 30 days following admission, even after adjustment on age and baseline severity (i.e., septic shock and PaO_2_:FiO_2_ (model 1; *p* = 0.046) or baseline SOFA score (model 2; *p* = 0.076)) (Supplementary Tables 1 and 2).

### Comparison of systemic and pulmonary concentrations of inflammatory mediators between non-COVID-19 and COVID-19 ARDS patients

**Fig. 1:**
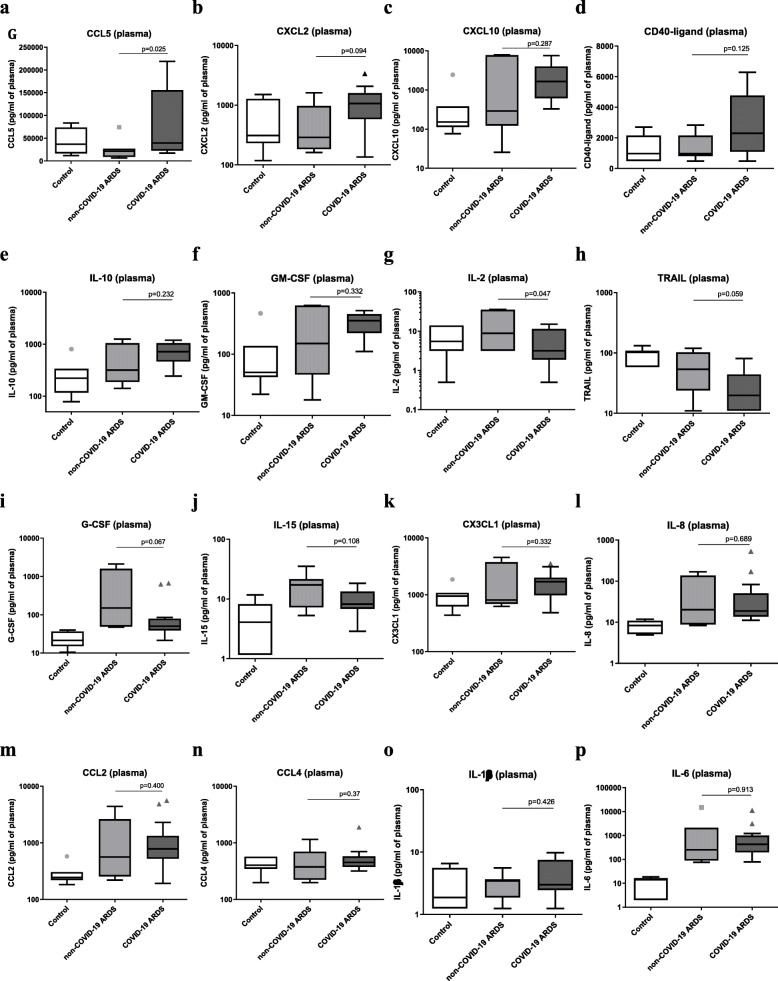
Boxplot graph depicting plasma concentration of cytokines. Plasma cytokines were measured in COVID-19 ARDS (*n* = 14), non-COVID-19 ARDS (*n* = 7), and control patients (*n* = 7). COVID-19 ARDS patients had significantly higher plasma concentrations of CCL5 and non-significantly higher plasma concentrations of CXCL2 (*p* = 0.09), CXCL10 (*p* = 0.29), CD40 ligand (*p* = 0.14), IL-10 (*p* = 0.23), and GM-CSF (*p* = 0.33) compared with non-COVID-19 ARDS patients. We observed lower concentrations of plasma IL-2 (*p* = 0.04), TRAIL (*p* = 0.055), and G-CSF (*p* = 0.06). The Mann-Whitney *U* test was used for comparison between non-COVID-19 and COVID-19 ARDS patients (Pneumochondrie study, 2019–2020)

Plasma cytokine levels are shown in Fig. 1 and Additional Table 3. COVID-19 ARDS patients had significantly higher plasma concentrations of CCL5 (*p* = 0.025) and non-significantly higher plasma concentrations of CXCL2 (*p* = 0.094), CXCL10 (*p* = 0.287), CD40 ligand (*p* = 0.125), IL-10 (*p* = 0.232), and GM-CSF (*p* = 0.332) compared with non-COVID-19 ARDS patients. At the same time, we observed lower concentrations of plasma IL-2 (*p* = 0.047), TRAIL (*p* = 0.059), and G-CSF (*p* = 0.067). Plasma CXCL10 concentration was independently associated with a greater number of ventilator-free days, after adjustment for the COVID-19 etiology, submission to non-invasive ventilation (NIV) prior to intubation, exposure to multiple antibiotics, CXCL2, CCL5, and CD40 ligand plasma concentrations (*p* = 0.049) (Table 2). Interestingly, CXCL10 levels were highly correlated with those of GM-CSF (*r* = 0.991; *p* < 0.0001) and IL-10 (*r* = 0.958; *p* < 0.0001).

**Table 2:** Plasma cytokines associated with the number of ventilator-free days in the 21 patients with ARDS (multivariate median logistic regression; pseudo-*R*^2^ = 0.432; *n* = 21)

Variables	Coefficient	***p*** value	95% confidence interval
COVID-19 etiology of ARDS	− 11.59787	0.128	− 27.003, 3.807256
Non-invasive mechanical ventilation	− 7.904059	0.221	− 21.19306, 5.384938
Antibiotic multitherapy	0.6376204	0.911	− 11.45826, 12.7335
[CXCL10]_plasma_	− 0.0027678	0.049	− 0.0055201, − 0.0000155
[CXCL2]_plasma_	0.0026861	0.527	− 0.0062382, 0.0116103
[CCL5]_plasma_	− 0.0001359	0.271	− 0.0003911, 0.0001193
[CD40 ligand]_plasma_	0.0055892	0.212	− 0.0036163, 0.0147947

**Fig. 2:**
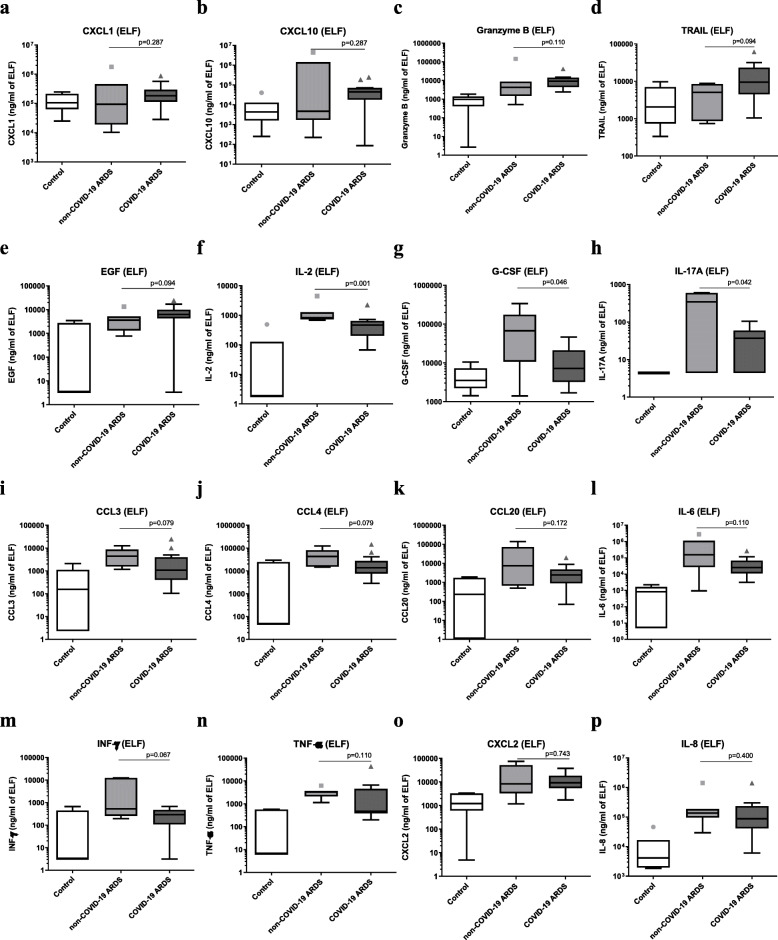
Boxplot graph depicting epithelial lining fluid (ELF) concentration of cytokines. Epithelial lining fluid concentration of the 16 main cytokines was measured in COVID-19 ARDS (*n* = 14), non-COVID-19 ARDS (*n* = 7), and control patients (*n* = 7). A trend toward higher ELF concentrations of CXCL1 (*p* = 0.29), CXCL10 (*p* = 0.29), granzyme B (*p* = 0.11), TRAIL (*p* = 0.09), and EGF (*p* = 0.09) is observed in COVID-19 patients. We also observed significantly lower ELF concentrations of IL-2 (*p* = 0.001), G-CSF (*p* = 0.046), and IL-17A (*p* = 0.037) and a trend toward lower concentrations of CCL3 (*p* = 0.08), CCL4 (*p* = 0.08), CCL20 (*p* = 0.17), IL-6 (*p* = 0.11), INF-γ (*p* = 0.06), and TNF-α (*p* = 0.11). The Mann-Whitney *U* test was used for comparison between non-COVID-19 and COVID-19 ARDS patients (Pneumochondrie study, 2019–2020)

Epithelial lining fluid cytokine levels are shown in Fig. 2 and Additional Table 4. A trend toward higher ELF concentrations of CXCL1 (*p* = 0.287), CXCL10 (*p* = 0.287), granzyme B (*p* = 0.110), TRAIL (*p* = 0.094), and EGF (*p* = 0.094) is observed in COVID-19 patients. We also observed significantly lower ELF concentrations of IL-2 (*p* = 0.001), G-CSF (*p* = 0.046), and IL-17A (*p* = 0.042) and a trend toward lower concentrations of CCL3 (*p* = 0.079), CCL4 (*p* = 0.079), CCL20 (*p* = 0.172), IL-6 (*p* = 0.110), INF-γ (*p* = 0.067), and TNF-α (*p* = 0.110). CXCL10 concentration in the epithelial lining fluid was independently associated with a greater number of ventilator-free days after adjusting for the COVID-19 etiology, submission to NIV prior to intubation, exposure to multiple antibiotics, TRAIL, TNF-α, and granzyme B ELF concentrations (*p* = 0.030) (Table 3).

**Table 3:** Epithelial lining fluid cytokines associated with the number of ventilator-free days in the 21 patients with ARDS (multivariate median logistic regression; pseudo-*R*^2^ = 0.449; *n* = 21)

Variables	Coefficient	***p*** value	95% confidence interval
COVID-19 etiology of ARDS	− 4.962124	0.391	− 17.04709, 7.122846
Non-invasive mechanical ventilation	− 7.301642	0.227	− 19.74284, 5.13956
Antibiotic multitherapy	6.531241	0.199	− 3.895659, 16.95814
[TRAIL]_ELF_	− 0.0002477	0.123	− 0.0005719, 0.0000764
[TNF-α]_ELF_	− 0.0001998	0.367	− 0.0006613, 0.0002616
[CXCL10]_ELF_	− 5.34e−06	0.030	− 0.0000101, − 5.96e−07
[Granzyme B]_ELF_	0.0000555	0.418	− 0.0000879, 0.0001988

### Systemic and pulmonary levels of cell-free mitochondrial DNA

**Fig. 3:**
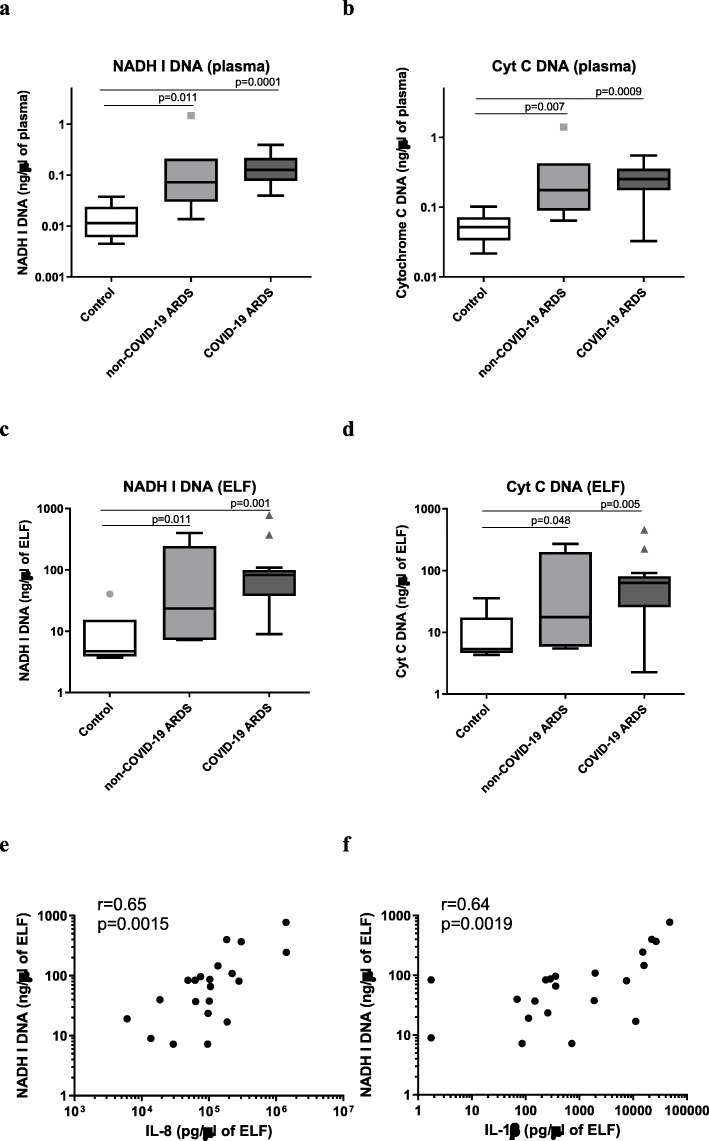
Boxplot graph depicting the concentration of cell-free mitochondrial DNA in the plasma and epithelial lining fluid (ELF). The concentration of cell-free mitochondrial DNA was measured in the plasma (NADH I (**a**) and cytochrome c (**b**) genes) and epithelial lining fluid (NADH I (**c**) and cytochrome c (**d**) genes) for COVID-19 ARDS (*n* = 14), non-COVID-19 ARDS (*n* = 7), and control patients (*n* = 7). The Kruskal-Wallis test with a false discovery rate post hoc multiple comparisons correction was used to adjust *p* values. Correlation between ELF concentrations of NADH I DNA and IL-8 (**e**) and IL-1β (**f**). Spearman correlations: *p < 0.05, **p < 0.01, and ***p < 0.01 between each cytokine and ELF concentration of NADH I mitochondrial DNA (Pneumochondrie study, 2019–2020)

We also addressed the magnitude and impact of mitochondrial DNA released into both the systemic and alveolar compartments during non-COVID-19 and COVID-19 ARDS. Plasma and ELF concentrations of mitochondrial DNA were significantly higher in ARDS patients, regardless of COVID-19 involvement (Fig. 3a–d). Interestingly, ELF concentrations of mtDNA were positively correlated with BALF cell count (*r* = 0.526; *p* = 0.014), as well as with concentrations of IL-8 (*r* = 0.647; *p* = 0.0015), IL-1β (*r* = 0.637; *p* = 0.0019), TGF-α (*r* = 0.582; *p* = 0.006), EGF (*r* = 0.532; *p* = 0.013), and CCL4 (*r* = 0.508; *p* = 0.019) (Fig. 3e–f; Additional Figure 1). Conversely, mtDNA concentrations were negatively correlated with CXCL10 (*r* = − 0.579; *p* = 0.006) and GM-CSF (*r* = − 0.519; *p* = 0.016) (Additional Figure 1). The same results were observed when only COVID-19 ARDS patients were considered (Additional Figure 2). However, neither plasma nor ELF mitochondrial DNA concentrations were correlated with the number of ventilator-free days.

### Epithelial lining fluid SARS-CoV-2 concentrations

**Fig. 4:**
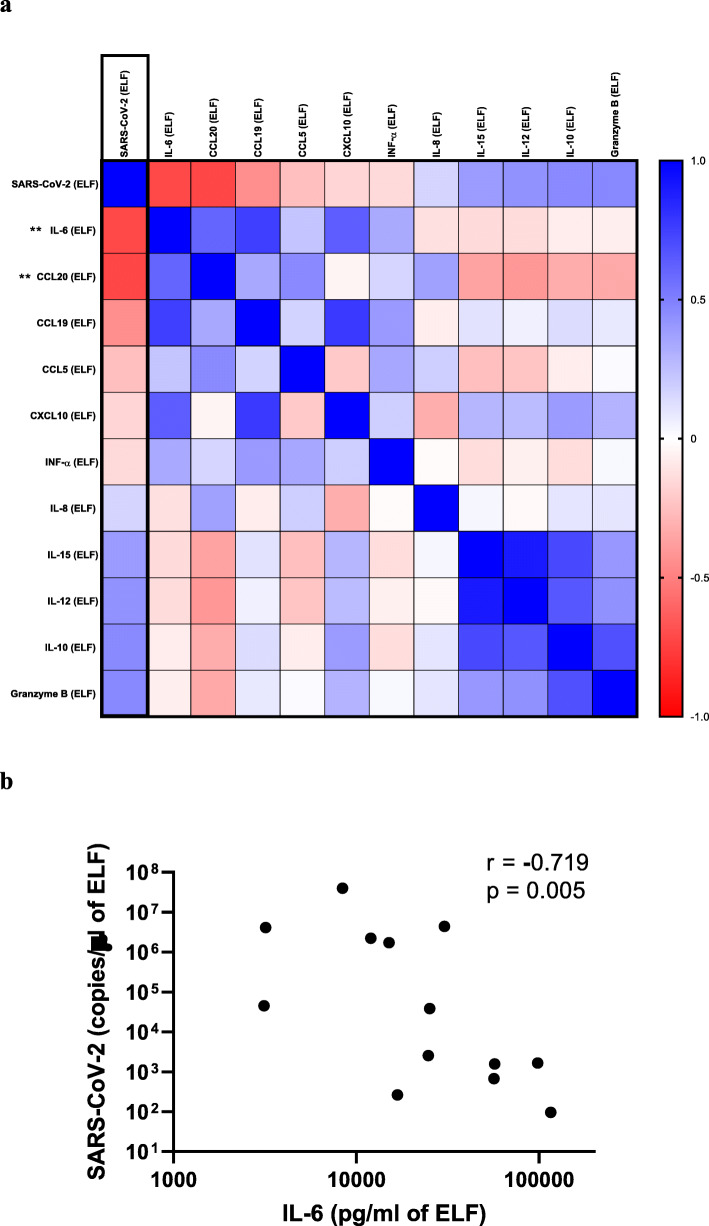
Correlations between epithelial lining fluid (ELF) SARS-CoV-2 viral load and ELF concentration of cytokines. Heatmap of the Spearman correlation (*r*) between epithelial lining fluid (ELF) concentrations of SARS-CoV-2 and ELF concentration of the main correlated cytokines for the 14 COVID-19 ARDS patients (**a**). Inverse correlation between the ELF concentrations of SARS-CoV-2 and IL-6 (**b**). Spearman correlations: *p < 0.05 and **p < 0.01 between each cytokine (our outcome) and ELF concentration of SARS-CoV-2 (Pneumochondrie study, 2019–2020)

Finally, SARS-CoV-2 concentrations were measured, and correlations with cytokine levels were sought in the alveolar compartment. First, the SARS-Cov-2 genome was detected in 13 COVID-19 ARDS patients with a median of 20,449 (IQR = 893–2,092,104) copies per microliter of ELF. There was a strong negative correlation with ELF IL-6 (*r* = − 0.719; *p* = 0.005) and CCL20 (*r* = − 0.723; *p* = 0.005), while no correlation was observed with ELF CXCL10 (*r* = − 0.165, *p* = 0.573) (Fig. 4a, b).

## Discussion

Our findings suggest that COVID-19 ARDS is associated with prolonged mechanical ventilation in comparison with non-COVID-19 ARDS patients, regardless of the baseline characteristics. This comprehensive evaluation of systemic and pulmonary immune response showed that the higher CXCL10 concentrations in both the systemic and alveolar compartments of patients with COVID-19 ARDS were associated with a longer duration of mechanical ventilation. Interestingly, alveolar concentrations of SARS-CoV-2 were not correlated with alveolar CXCL10 concentration. Finally, in both COVID-19 and non-COVID-19 patients, higher mitochondrial DNA concentrations in the plasma and ELF compartment were highly correlated with alveolar inflammation, as assessed by BALF cell count and ELF IL-8 and IL-1β concentrations. This result highlights the key role of mitochondrial alarmins in ARDS and VILI.

ARDS is the leading cause of death during COVID-19. Given the increasing number of cases, and especially the protracted duration of ARDS, attention was rapidly focused on the number of required ICU beds, ventilators, and intensivists [19]. However, COVID-19-associated ARDS, despite meeting the Berlin Definition, is now considered “atypical” with peculiar features such as the discrepancy between the relatively well-preserved lung mechanics and the depth of hypoxemia [20]. Our findings support these observations since we show that patients with COVID-19 ARDS received prolonged mechanical ventilation when compared to non-COVID-19 ARDS patients, regardless of baseline severity.

In the most severe forms of SARS-CoV-2, common features including systemic cytokine storm (including macrophage activation syndrome), organizing diffuse alveolar damage (acute fibrinous and organizing pneumonia (AFOP)) with excessive immune cells infiltration into the lung (in particular T cell infiltration), and thrombosis have been reported, but these features have also been observed in patients with SARS-CoV-1 infection and Middle East respiratory syndrome (MERS) [9, 21–23]. We assume that this atypical dysregulated immune response may cause the lung immunopathology that leads to the protracted and inflammatory nature of COVID-19 ARDS. Since there is no available effective direct anti-viral treatment so far, mitigating the “cytokine storm” through immune modulation is a promising therapeutic avenue. However, it is worth noting that most of the currently tested immunomodulatory agents (especially anti-IL-6, anti-IL-1β, and anti-TNFα) have been put forward despite gaps in our understanding of the immune response behind COVID-19 ARDS, especially within the pulmonary compartment. However, assessing the alveolar compartment is challenging in severe ARDS patients. To the best of our knowledge, there is currently no published study comparing cytokine concentrations in bronchoalveolar lavage fluid obtained from either COVID-19 or non-COVID-19 ARDS patients, and basically, data regarding inflammation within the alveolar space of the former patients are sparse [24]. We report herein new insights into this issue.

We show that plasma CXCL2, CXCL10, CCL5, CD40 ligand, IL-10, and GM-CSF concentrations and ELF CXCL1, CXCL10, granzyme B, TRAIL, and EGF concentrations were found in greater concentrations in COVID-19 than in non-COVID-19 ARDS patients. Interestingly, almost all of these mediators are chemokines or cytokines involved in either recruitment or activation of T lymphocytes (CCL5, CXCL10) and/or monocytes/macrophages (GM-CSF). Others are associated with anti-inflammation (IL-10 and TRAIL) or endothelial dysfunction (CD40 ligand, EGF). Among them, we found that CXCL10 was the most likely to account for the protracted nature of COVID-19 ARDS in both the systemic and the alveolar compartments. CXCL10 (or INF-γ-induced protein (IP-10)) is secreted upon INF-γ stimulation by various cell types (endothelial cells, fibroblasts, monocytes/macrophages, and T lymphocytes) and promotes chemoattraction for activated T lymphocytes, natural killer cells, and monocytes through CXCR3 [25]. Interestingly, plasma concentrations of CXCL10 have been previously reported at high levels in SARS-CoV-1 [26], respiratory syncytial [27], and influenza infections [28], and significantly associated with a higher risk of death in ARDS associated with A/H1N1 influenza infection [29]. Yang et al. recently reported that among 44 inflammatory mediators measured in COVID-19 patients, the plasma concentration of CXCL10 was highly associated with disease severity, especially the Murray score, and could independently predict COVID-19 disease progression [9]. Interestingly, Ichikawa et al. showed that the CXCL10-CXCR3 signaling pathway was critical in viral and non-viral ARDS pathogenesis. Moreover, they showed that lung injury could be prevented, and therefore, survival improved, when the CXCL10-CXCR3 axis was blocked [30]. Since our results emphasize the involvement of the CXCL10-CXCR3 signaling axis in the pathogenesis of the most severe forms of COVID-19 infection, this axis represents a potential therapeutic target. Corticosteroids that showed beneficial effects in the most severe forms of COVID-19 [31] may act upstream from CXCL10 through inhibition of the Th1 pathway. However, specifically blocking CXCL10 (e.g., eldelumab/MDX-1100) or CXCR3 may be a promising therapeutic approach. Interestingly, Lev et al. reported that upon the administration of corticosteroid, a significant decrease of CXCL10 levels was observed in COVID-19 patients [32]. In addition, if validated in larger cohorts, plasma CXCL10 measurement could be helpful in predicting the risk of prolonged MV.

Since plasma CXCL-10 and GM-CSF were strongly correlated (*r* = 0.991, *p* < 0.0001), the latter was also highly associated with a longer duration of mechanical ventilation. The most severe forms of COVID-19 were associated with macrophage activation syndrome, characterized by a fulminant hypercytokinemia with multiorgan failure [23]. GM-CSF has recently emerged as a potential therapeutic target, since it plays a pivotal role in initiation (monocyte activation and macrophage transformation) and perpetuation of the immune response and could represent the link between T cell-driven acute pulmonary inflammation and self-amplifying cytokine loop leading to monocyte/macrophage activation [33, 34]. Interestingly, an anti-GM-CSF therapy (lenzilumab) has been previously evaluated in severe COVID-19 and associated with improved clinical outcomes, oxygen requirement, and cytokine release [35].

Still, a cautious approach is required since any immune modulation is likely to alter the body’s antimicrobial defenses. This point is particularly relevant, since deep dysfunctions of the myeloid and lymphoid responses have also been described in COVID-19 patients, with a decrease of HLA-DR expression on monocytes [23] and T cell exhaustion [36]. Interestingly, no correlation was found between the alveolar viral load of SARS-CoV-2 and ELF CXCL10, suggesting that activation of the CXCL10-CXCR3 axis may play a role in the dysregulated immune response independently from viral clearance. Thus, its blockage might not compromise the host’s ability to bring the virus under control. In contrast, we observed a strong negative correlation between ELF concentrations of SARS-CoV-2 and IL-6, suggesting that IL-6 production within the alveolar compartment is associated with effective viral clearance. In addition, dampening inflammation in a context of high immune suppression level is not always a hazardous route. In the setting of chimeric antigen receptor T (CAR-T) cell therapy, GM-CSF inhibition reduces cytokine release syndrome and neuro-inflammation but enhances antitumoral CART-T cell function [37]. Moreover, the IL-6 blocker partially rescues immune dysregulation caused by SARS-CoV-2 [23]. Nevertheless, as advocated for sepsis [38], immune profiling (including CXCL10 measurement) may serve for the selection of patients that could be eligible for immunotherapy.

Finally, it is worth noting that higher viral alveolar loads were associated with more severe ARDS in terms of blood oxygenation and remote organ failure [39], highlighting the need for drugs likely to prevent COVID-19 replication in addition to therapies targeting host response.

In addition to our findings regarding the CXCL10-CXCR3 axis, we observed that the released amount of mediators involved in chemotaxis and/or activation of PMNs (i.e., IL-8, IL-1β, IL-6, and TNF-α) was similar when ARDS was caused by SARS-CoV-2. Moreover, alveolar cell counts and ELF concentrations of IL-8 and Il-1β were highly correlated with the release of mitochondrial DNA, whose levels were significantly increased in both the systemic and pulmonary compartments of ARDS patients, regardless of etiology. It is worth noting that cell-free mitochondrial DNA release can elicit neutrophil-mediated lung injury through the promotion of IL-8 and Il-1β secretion by activation of the TLR-9 (Toll-like receptor 9) and the NLRP-3 (NOD-like receptor pyrin domain 3) inflammasome pathways, respectively [15, 40]. This finding suggests that systemic and pulmonary immune response could be triggered in part by the release of endogenous mediators originating from injured alveolar cells. This reaction may be subsequent to a two-hit lung injury (i.e., infection of alveolar epithelial cells and ventilator-induced lung injury), as suggested by previous clinical and experimental findings from our group [10, 11].

Surprisingly, we observed a trend toward lower ELF concentrations of IL-6 in COVID-19 patients compared with non-COVID-19 ARDS patients (*p* = 0.11), thus challenging the interest of anti-IL-6 therapies in severe COVID-19 pneumonia. These results are in line with retrospective observations by Sinha et al., showing that plasma IL-6 concentrations were lower in the severe form of COVID-19 as compared to those reported in ARDS from another origin and arguing that the term “cytokine storm” may be misleading in COVID-19 ARDS [41].

Finally, it is worth noting that early bacterial coinfection was unlikely in COVID-19 ARDS patients from our cohort, in accordance with previously published data [42, 43]. One could argue that previous exposure to antibiotics has led to false-negative results. More interestingly, it ascertains the safety of introducing new therapies (i.e., including those targeting CXCL-10) likely to dampen the host inflammatory response at this stage of the viral disease. This statement has to be mitigated by the fact that VAP complicated ARDS in 71% of the COVID-19 patients, as compared to 0% in patients with ARDS from another origin. Although the longer duration of MV could account at least in part for such an obvious difference, one cannot exclude that VAP occurrence reflected exhaustion of the lung immune defense following the strong activation of the CXCL10-CXCR3 axis. Moreover, the impact of blocking this critical pathway remains uncertain regarding the risk of subsequent bacterial infection.

Our study has some limitations. First, the origin of non-COVID-19 ARDS was mainly Gram-negative bacteria, which is not representative of all ARDS of pulmonary origin. As a result of the difficulty in obtaining BALF, the small sample size prevented us from performing a mortality analysis and resulted in a lack of power for some comparisons. As a result, our findings should be taken cautiously, and new studies are required in order to ascertain our hypothesis. However, pulmonary compartment assessment remains challenging, and there is currently no published data comparing BALF in COVID-19 and non-COVID-19 ARDS patients. Moreover, the criteria of inclusion of our control group remain questionable, and non-infected ICU patients under MV would have been preferred but not possible given ethical concerns related to the fiberoptic procedure, which could not be considered as part of standard care. One can also argue that in the context of such an emerging disease, some treatments likely to influence the release of cytokines could have been given to the COVID-19 patients prior to the study inclusion. However, such treatments (i.e., hydrocortisone and hydroxychloroquine) were administrated prior to blood and BALF collection in a small number of patients. Moreover, the time elapsed between treatment administration and sampling was quite short, making unlikely any impact on cytokine concentrations (Additional Table 5). Finally, the specific impact of mechanical ventilation on pulmonary immune response cannot be evaluated since patients with less severe disease who did not require MV were not included.

## Conclusions

This study provides new insights into the peculiar pathogenesis of COVID-19 ARDS. First, CXCL10 may represent the dysregulated immune response that drives the duration of MV in COVID-19 ARDS patients. CXCL10 appears to be a potential biomarker for the duration of MV, and the CXCL10-CXCR3 signaling axis may be a potential therapeutic target in COVID-19 ARDS. This target seems all the more interesting since no correlation was found between alveolar concentrations of CXCL10 and SARS-CoV-2 clearance. Apart from those particular features, COVID-19 and non-COVID-19 ARDS share the well-known involvement of both IL-8 and IL-1β within the airway, potentially driven by the release of endogenous mediators originating from injured alveolar cells (e.g., mitochondrial alarmins).

## Supplementary information

**Additional file 1: Table 1.** Model 1: factors associated with the number of ventilator-free days in the 21 patients with ARDS (multivariate median logistic regression; pseudo R^2^ = 0.237; *n*=21, Pneumochondrie study, 2019-2020). **Table 2.** Model 2: factors associated with the number of ventilator-free days in the 21 patients with ARDS (multivariate median logistic regression; pseudo R^2^ = 0.223; n=21, Pneumochondrie study, 2019-2020). **Table 3.** Plasma concentrations of cytokines. **Table 4.** Epithelial lining fluid concentrations of cytokines. **Figure 1.** Heatmap of the Spearman correlation (r) between epithelial lining fluid (ELF) concentrations of mitochondrial DNA (NADH I and Cytochrome C), ELF concentration of cytokines, and outcomes for the 21 ARDS patients. Spearman correlations**:** *p*<0.05 *; *p*<0.01 ** between each cytokine and the ELF concentration of NADH I mitochondrial DNA. **Figure 2**. Heatmap of the Spearman correlation (r) between the epithelial lining fluid (ELF) concentrations of mitochondrial DNA (NADH I and Cytochrome C), the ELF concentration of cytokines and outcomes for the 14 COVID-19 ARDS patients (A). Spearman correlations**:** p<0.05 *; p<0.01 ** between each cytokine and the ELF concentration of NADH I mitochondrial DNA.

## Data Availability

All data are available on demand.

## Abbreviations

ARDS: Acute respiratory distress syndrome
BAL: Bronchoalveolar lavage
BALF: Bronchoalveolar lavage fluid
CCL: C-C motif chemokine ligand
COVID-19: Coronavirus disease 2019
CXCL: C-X-C motif chemokine ligand
CXCR3: CXC chemokine receptor 3
DNA: Deoxyribonucleic acid
ECMO: Extracorporeal membrane oxygenation
ELF: Epithelial lining fluid
EGF: Epidermal growth factor
FGF: Fibroblast growth factor
FLT3L: FMS-like tyrosine kinase 3 ligand
G-CSF: Granulocyte colony-stimulating factor
GM-CSF: Granulocyte-macrophage colony-stimulating factor
ICU: Intensive care medicine
IFN: Interferon
IL: Interleukin
IP-10: INF-γ-induced protein
IQR: Interquartile range
MV: Mechanical ventilation
NADH I: Nicotinamide adenine dinucleotide I
NLRP3: NOD-like receptor pyrin domain 3
PaO_2_:FiO_2_: Arterial pressure of oxygen/oxygen inspiratory fraction
PD-L1: Programmed death-ligand 1
PDGF: Platelet-derived growth factor
RDRP: RNA-dependent RNA polymerase
RT-PCR: Reverse transcriptase polymerase chain reaction
SAPS II: Simplified Acute Physiology Score II
SARS-CoV-2: Severe acute respiratory syndrome coronavirus 2
SOFA: Sequential Organ Failure Assessment
TGF: Transforming growth factor
TLR-9: Toll-like receptor 9
TNF: Tumor necrosis factor
TRAIL: TNF-related apoptosis-inducing ligand
VAP: Ventilator-acquired pneumonia
VEGF: Vascular endothelial growth factor
