# Correction to: CXCL10 could drive longer duration of mechanical ventilation during COVID-19 ARDS

**DOI:** 10.1186/s13054-021-03559-9

**Published:** 2021-04-13

**Authors:** Mathieu Blot, Marine Jacquier, Ludwig-Serge Aho Glele, Guillaume Beltramo, Maxime Nguyen, Philippe Bonniaud, Sebastien Prin, Pascal Andreu, Belaid Bouhemad, Jean-Baptiste Bour, Christine Binquet, Lionel Piroth, Jean-Paul Pais de Barros, David Masson, Jean-Pierre Quenot, Pierre-Emmanuel Charles, Pneumochondrie Study Group

**Affiliations:** 1 Infectious Diseases Department, Dijon Bourgogne University Hospital, Dijon, France; 2 INSERM, LNC UMR 1231, FCS Bourgogne-Franche Comté LipSTIC LabEx, Dijon, France; 3 Department of Pneumology, Dijon Bourgogne University Hospital, Dijon, France; 4 Anesthesiology and Critical Care Department, Dijon Bourgogne University Hospital, Dijon, France; 5 Laboratory of Virology, Dijon Bourgogne University Hospital, Dijon, France; 6 INSERM, CIC1432, Clinical Epidemiology Unit, Dijon Bourgogne University Hospital, Clinical Investigation Center, Clinical Epidemiology/Clinical Trials Unit, Dijon, France; 7 Lipidomic Analytic Unit, University Bourgogne Franche-Comté, Bâtiment B3, Bvd. Maréchal de Lattre de Tassigny, 21000 Dijon, France; 8 Laboratory of Clinical Chemistry, Dijon Bourgogne University Hospital, Dijon, France; 9 Department of Intensive Care, Dijon Bourgogne University Hospital, Dijon, France; 10 Epidemiology and Hospital Hygiene Department, Dijon Bourgogne University Hospital, Dijon, France

## Correction to: *Crit Care* (2020) 24:632 https://doi.org/10.1186/s13054-020-03328-0

Following publication of the original article [1], the authors identified an error in the affiliations.

All the changes requested are implemented in this correction and the original article [1] has been corrected.
