# Supplementary material for: CXCL10 could drive longer duration of mechanical ventilation during COVID-19 ARDS
Source: Crit Care. 2020 Nov 2;24:632. doi: 10.1186/s13054-020-03328-0 (PMC7604548; doi:10.1186/s13054-020-03328-0)
Supplement: Supplementary file 1 — Additional file 1: Table 1. Model 1: factors associated with the number of ventilator-free days in the 21 patients with ARDS (multivariate median logistic regression; pseudo R2 = 0.237; n=21, Pneumochondrie study, 2019-2020). Table 2. Model 2: factors associated with the number of ventilator-free days in the 21 patients with ARDS (multivariate median logistic regression; pseudo R2 = 0.223; n=21, Pneumochondrie study, 2019-2020). Table 3. Plasma concentrations of cytokines. Table 4. Epithelial lining fluid concentrations of cytokines. Figure 1. Heatmap of the Spearman correlation (r) between epithelial lining fluid (ELF) concentrations of mitochondrial DNA (NADH I and Cytochrome C), ELF concentration of cytokines, and outcomes for the 21 ARDS patients. Spearman correlations: p<0.05 *; p<0.01 ** between each cytokine and the ELF concentration of NADH I mitochondrial DNA. Figure 2. Heatmap of the Spearman correlation (r) between the epithelial lining fluid (ELF) concentrations of mitochondrial DNA (NADH I and Cytochrome C), the ELF concentration of cytokines and outcomes for the 14 COVID-19 ARDS patients (A). Spearman correlations: p<0.05 *; p<0.01 ** between each cytokine and the ELF concentration of NADH I mitochondrial DNA. [file 13054_2020_3328_MOESM1_ESM.docx]

**CXCL10 could drive longer duration of mechanical ventilation during COVID-19 ARDS**

**Additional files (Pneumochondry study 2019-2020)**

Mathieu Blot, MD, PhD^1,2^, Marine Jacquier, MSC^1,3^, Ludwig-Serge Aho Glele, MD^4^, Guillaume Beltramo, MD^5^, Maxime Nguyen, MD^2,6^, Philippe Bonniaud, MD, PhD^5^, Sebastien Prin, MD^3^, Pascal Andreu, MD^3^, Belaid Bouhemad, MD, PhD^2,6^, Jean-Baptiste Bour, Pharm^7^, Christine Binquet, MD, PhD^8^, Lionel Piroth, MD, PhD^1,2^, Jean-Paul Pais de Barros, PhD^2,9^, David Masson, Pharm, PhD^2,10^, Jean-Pierre Quenot, MD, PhD^2,3,8^, Pierre-Emmanuel Charles, MD, PhD^2,3^. Pneumochondrie study group.

^1^ Infectious Diseases Department, Dijon Bourgogne University Hospital, Dijon, France

^2^ Univ. Bourgogne Franche-Comté, LNC UMR1231, F-21000, Dijon, France ; INSERM, LNC UMR 1231, F-21000, Dijon, France ; FCS Bourgogne-Franche Comté, LipSTIC LabEx, F-21000, Dijon, France.

^3^ Department of Intensive Care, Dijon Bourgogne University Hospital, Dijon, France

^4^ Epidemiology and Hospital Hygiene Department, Dijon Bourgogne University Hospital, Dijon, FR-21079.

^5^ Department of pneumology, Dijon Bourgogne University Hospital, Dijon, France

^6^ Anesthesiology and Critical Care Department, Dijon Bourgogne University Hospital, Dijon, France

^7^ Laboratory of virology, Dijon Bourgogne University Hospital, Dijon, France

^8^ INSERM, CIC1432, Clinical Epidemiology unit, Dijon, France; Dijon Bourgogne University Hospital, Clinical Investigation Center, Clinical Epidemiology/Clinical trials unit, Dijon, France.

^9^ Lipidomic analytic unit, Univ. Bourgogne Franche-Comté, Bâtiment B3, Bvd Maréchal de Lattre de Tassigny, 21000, Dijon, France.

^10^ Laboratory of Clinical Chemistry, Dijon Bourgogne University Hospital, Dijon, France

**Additional Table 1.** Model 1: factors associated with the number of ventilator-free days in the 21 patients with ARDS (multivariate median logistic regression; pseudo R² = 0.237; n=21, Pneumochondrie study, 2019-2020).

| **Variables** | **Coefficient** | ***p-value*** | **95% confiance intervalle** |
| --- | --- | --- | --- |
| Age | -0.024938 | 0.917 | -0.526595 0.4767189 |
| COVID-19 etiology | -15.87785 | 0.046 | -31.41698 -0.3387173 |
| Septic shock | -8.95995 | 0.204 | -23.2926 5.372698 |
| PaO_2_:FiO_2_ ratio | -0.0261339 | 0.678 | -0.1572961 0.1050283 |

**Additional Table 2.** Model 2: factors associated with the number of ventilator-free days in the 21 patients with ARDS (multivariate median logistic regression; pseudo R² = 0.223; n=21, Pneumochondrie study, 2019-2020).

| **Variables** | **Coefficient** | ***p-value*** | **95% confiance intervalle** |
| --- | --- | --- | --- |
| Age | -0.125 | 0.518 | -0.5240105 0.2740105 |
| COVID-19 etiology | -11.75 | 0.076 | -24.87157 1.371568 |
| Baseline SOFA score | -0.375 | 0.668 | -2.186286 1.436286 |

**Additional Table 3. Plasma concentrations of cytokines**

|  | **Study groups** | | | | | |  |
| --- | --- | --- | --- | --- | --- | --- | --- |
|  | **Control** | | **non-COVID-19 ARDS** | | **COVID-19 ARDS** | |  |
|  | **n=7** | | **n=7** | | **n=14** | |  |
|  | Median | IQR | Median | IQR | Median | IQR | p* |
| **sCD40-Ligand, pg/ml** | 974.24 | 606.76-1,945.83 | 974.24 | 892.4-1,684.63 | 2,296.29 | 1,132.89-4,661.25 | 0.125 |
| **EGF, pg/ml** | 38.94 | 14.13-43.56 | 28.74 | 18.22-44.12 | 89.34 | 36.17-143.58 | 0.044 |
| **CCL11, pg/ml** | 202.71 | 149.26-341.91 | 147.31 | 134.95-229.82 | 217.86 | 144.13-268.83 | 0.636 |
| **FGF, pg/ml** | 1.14 | 1.14-6.82 | 1.14 | 1.14-25.9 | 17.31 | 1.14-36.25 | 0.657 |
| **FLT3L, pg/ml** | 175.37 | 163.25-179.36 | 155.1 | 123.05-184.87 | 201.05 | 163.24-271.11 | 0.108 |
| **CX3CL1, pg/ml** | 946.69 | 762.59-1061.1 | 808.99 | 716.84-2367.99 | 1,699.42 | 1,038.19-1,847.52 | 0.332 |
| **G-CSF, pg/ml** | 21.3 | 15.67-33.01 | 150.33 | 54.35-999.22 | 50.395 | 39.24-73.25 | 0.067 |
| **GM-CSF, pg/ml** | 50.28 | 46.34-119.88 | 148.99 | 78.57-464.08 | 352.64 | 230.31-441.25 | 0.332 |
| **Granzyme B, pg/ml** | 28.85 | 15.68-29.55 | 23.16 | 15.16-67.84 | 49.615 | 29.67-57.76 | 0.970 |
| **CXCL1, pg/ml** | 99.85 | 92.79-131.89 | 99.85 | 88.96-145.24 | 119.77 | 101.5-256.08 | 0.296 |
| **CXCL2, pg/ml** | 311.84 | 258.69-868.54 | 288.64 | 208.00-795.08 | 1,065.06 | 584.85-1,548.22 | 0.094 |
| **INF-α, pg/ml** | 7.62 | 4.34-15.57 | 6.83 | 1.42-8.39 | 6.82 | 4.55-13.16 | 0.475 |
| **INF-γ, pg/ml** | 5.54 | 3.89-20.45 | 11.72 | 5.46-28.03 | 3.08 | 2.24-20.52 | 0.484 |
| **IL1-α, pg/ml** | 19.93 | 12.05-32.58 | 15.93 | 8.79-21.81 | 16.45 | 13.78-25.77 | 0.432 |
| **IL1-β, pg/ml** | 1.87 | 1.25-5.09 | 3.55 | 2.17-3.55 | 3.005 | 2.46-7.38 | 0.426 |
| **IL1-RA, pg/ml** | 497.95 | 448.88-685.31 | 9,119.67 | 2,431.77-9,295.53 | 2,799.28 | 1,964.62-5,085.31 | 0.314 |
| **IL-2, pg/ml** | 5.49 | 3.75-10.86 | 8.8 | 5.98-33.43 | 3.15 | 1.89-10.25 | 0.047 |
| **IL-4, pg/ml** | 0.70 | 0.10-2.04 | 0.41 | 0.10-0.84 | 0.7 | 0.41-1.18 | 0.326 |
| **IL-6, pg/ml** | 15.12 | 3.05-15.12 | 249.74 | 127.04-1,673.82 | 427.31 | 221.75-831.59 | 0.913 |
| **IL-7, pg/ml** | 5.2 | 3.95-8.87 | 11.27 | 6.29-17.69 | 10.67 | 8.27-13.48 | 0.940 |
| **IL-8, pg/ml** | 8.32 | 6.38-10.10 | 20.12 | 9.03-127.96 | 18.75 | 14.53-38.48 | 0.689 |
| **IL-10, pg/ml** | 221.39 | 134.93-317.00 | 317.12 | 226.28-834.23 | 715.615 | 466.13-1,009.97 | 0.232 |
| **IL-12, pg/ml** | 2.93 | 2.93-13.01 | 10.54 | 8.04-22.72 | 10.53 | 2.93-19.72 | 0.469 |
| **IL-13, pg/ml** | 89.6 | 74.02-122.52 | 73.51 | 37.26-85.79 | 63.88 | 41.05-79.86 | 0.793 |
| **IL-15, pg/ml** | 4.09 | 2.63-6.33 | 17.21 | 10.21-19.90 | 8.17 | 7.32-12.28 | 0.108 |
| **IL-17A, pg/ml** | 6.61 | 4.60-10.61 | 4.81 | 4.39-14.55 | 4.39 | 4.39-6.06 | 0.326 |
| **IL-33, pg/ml** | 19.75 | 12.35-30.09 | 23.26 | 17.95-27.55 | 23.23 | 13.36-32.17 | 0.881 |
| **CXCL10, pg/ml** | 152.29 | 126.50-313.62 | 290.47 | 138.42-4,449.58 | 1,652.27 | 658.73-2,949.30 | 0.287 |
| **CCL2, pg/ml** | 245.34 | 228.54-288.73 | 562.04 | 322.49-1618.77 | 780.81 | 575.70-982.44 | 0.400 |
| **CCL3, pg/ml** | 26.57 | 12.32-35.04 | 29.27 | 21.12-56.21 | 30.28 | 22.28-43.66 | 1.000 |
| **CCL4, pg/ml** | 402.63 | 348.25-521.67 | 376.42 | 233.93-607.08 | 450.5 | 382.97-566.14 | 0.370 |
| **CCL20, pg/ml** | 37.33 | 21.44-49.36 | 380.56 | 101.00-617.80 | 103.02 | 57.32-366.92 | 0.224 |
| **CCL19, pg/ml** | 159.19 | 143.70-376.65 | 671.93 | 382.82-1,492.18 | 485.09 | 307.98-631.42 | 0.322 |
| **PDGF-AA, pg/ml** | 2632.41 | 1,998.07-3,870.36 | 2,037.99 | 1,517.92-3,801.08 | 2,367.99 | 1,845.63-5,179.30 | 0.478 |
| **PDGF-AB/BB, pg/ml** | 531.45 | 448.70-1,072.55 | 682.6 | 450.39-869.73 | 1,173.56 | 664.92-2,319.78 | 0.110 |
| **PD-L1, pg/ml** | 228.37 | 146.41-240.21 | 156.2 | 131.56-462.82 | 287.12 | 198.51-344.97 | 0.601 |
| **CCL5, pg/ml** | 36,752.12 | 23,257.10-63,589.01 | 21,494.95 | 90,37.68-25,396.88 | 39,638.81 | 23,626.37-143,579.97 | 0.025 |
| **TGF-α, pg/ml** | 20.5 | 10.92-26.46 | 27.95 | 26.48-29.40 | 15.06 | 10.99-17.42 | 0.050 |
| **TNF-α, pg/ml** | 20.49 | 17.67-38.93 | 34.96 | 24.16-80.59 | 38.48 | 15.09-51.29 | 0.502 |
| **TRAIL, pg/ml** | 102.59 | 65.07-105.79 | 53.56 | 36.34-80.41 | 19.8 | 6.56-41.57 | 0.059 |
| **VEGF, pg/ml** | 133.31 | 121.04-158.96 | 199.84 | 190.59-439.16 | 315.15 | 199.82-481.67 | 0.737 |
| Notes: * Comparison between non-COVID-19 and COVID-19 ARDS groups (Mann-Whitney test) | | | | |  |  |  |

**Additional Table 4. Epithelial lining fluid concentrations of cytokines**

|  | **Study groups** | | | | | |  |
| --- | --- | --- | --- | --- | --- | --- | --- |
|  | **Control** | | **non-COVID-19 ARDS** | | **COVID-19 ARDS** | |  |
|  | **n=7** | | **n=7** | | **n=14** | |  |
|  | Median | IQR | Median | IQR | Median | IQR | p* |
| **sCD40-Ligand, pg/ml** | 243.56 | 243.56-6,120.57 | 32,276.19 | 22,899.11-41,873.41 | 16,596.42 | 9,979.45-29,722.36 | 0.197 |
| **EGF, pg/ml** | 3.335 | 3.34-1,610.09 | 3591.78 | 1688.51-4,573.17 | 6,223.62 | 4,381.83-9,202.47 | 0.094 |
| **CCL11, pg/ml** | 982.89 | 752.71-2,009.34 | 841.52 | 662.88-2,224.23 | 1,187.26 | 720.39-1,589.17 | 1.000 |
| **FLT3L, pg/ml** | 401.74 | 10.52-1626.13 | 4,254.13 | 3,264.63-5,992.51 | 3261.79 | 1,960.01-5364.46 | 0.197 |
| **CX3CL1, pg/ml** | 7501.10 | 6,436.20-9,452.67 | 12,349.932 | 9,597.91-23,964.51 | 16,444.49 | 10,551.05-30,593.49 | 0.856 |
| **G-CSF, pg/ml** | 3527.69 | 2381.47-5961.22 | 67,324.51 | 18,227.22-133,990.18 | 7,210.513 | 3,998.90-17,792.07 | 0.046 |
| **GM-CSF, pg/ml** | 1,493.21 | 380.81-3,481.93 | 2,538.56 | 1,567.34-8,929.94 | 4,350.692 | 2,382.70-10,611.21 | 0.488 |
| **Granzyme B, pg/ml** | 973.642 | 639.64-1,181.10 | 4,328.116 | 1,831.32-6,515.90 | 9,242.203 | 5,102.00-13,992.42 | 0.110 |
| **CXCL1, pg/ml** | 105,736.39 | 77,616.30-181,308.54 | 93,866.61 | 39,555.27-298,241.17 | 183,014.50 | 122,305.92-261,058.11 | 0.287 |
| **CXCL2, pg/ml** | 1,224.62 | 786.77-2,295.80 | 8,333.23 | 4,856.90-44,912.90 | 9,226.36 | 5,903.76-16,945.20 | 0.743 |
| **INF-α, pg/ml** | 2.29 | 2.29-2.29 | 73.08 | 28.40-195.33 | 46.36 | 25.63-75.21 | 0.430 |
| **INF-γ, pg/ml** | 3.12 | 3.12-347.56 | 529.10 | 321.88-7,207.11 | 290.07 | 124.28-434.70 | 0.067 |
| **IL1-α, pg/ml** | 5.1 | 5.10-95.25 | 1,778.30 | 394.22-2,498.51 | 260.27 | 144.00-511.84 | 0.197 |
| **IL1-β, pg/ml** | 159.40 | 37.77-351.14 | 11,165.108 | 490.02-15,537.31 | 323.92 | 122.27-1,941.96 | 0.218 |
| **IL1-RA, pg/ml** | 98,319.73 | 57,838.98-112,184.95 | 265,380.82 | 174,233.72-508,308.41 | 186,720.90 | 64,960.94-311,532.5 | 0.224 |
| **IL-2, pg/ml** | 1.79 | 1.79-92.04 | 826.05 | 767.94-1,061.88 | 466.42 | 233.22-625.15 | 0.001 |
| **IL-3, pg/ml** | 8.28 | 8.28-465.71 | 307.232 | 206.47-589.52 | 125.64 | 8.28-194.20 | 0.070 |
| **IL-5, pg/ml** | 3.20 | 3.20-3.20 | 156.75 | 74.87-273.78 | 55.19 | 11.91-134.76 | 0.033 |
| **IL-6, pg/ml** | 827.54 | 207.26-1,491.78 | 154,813.98 | 29,591.16-670,473.11 | 25,028.09 | 12,791.48-57,075.16 | 0.110 |
| **IL-7, pg/ml** | 89.92 | 66.59-106.95 | 66.23 | 48.73-94.69 | 91.68 | 70.88-118.34 | 0.400 |
| **IL-8, pg/ml** | 4,122.60 | 2,447.19-10,580.25 | 136,389.27 | 96,326.94-185,611.61 | 88,336.74 | 52,127.41-192,684.91 | 0.400 |
| **IL-10, pg/ml** | 1,664.843 | 394.46-6,897.23 | 7,003.03 | 4,374.25-13,268.71 | 9,484.708 | 6,673.40-19,640.73 | 0.535 |
| **IL-12, pg/ml** | 413.88 | 202.30-534.64 | 405.58 | 298.42-477.08 | 293.06 | 138.41-435.71 | 0.455 |
| **IL-13, pg/ml** | 14.61 | 14.61-14.61 | 371.49 | 14.61-673.89 | 273.04 | 14.61-597.96 | 0.878 |
| **IL-15, pg/ml** | 156.46 | 124.24-225.36 | 211.28 | 114.72-429.12 | 108.46 | 92.20-189.61 | 0.197 |
| **IL-17A, pg/ml** | 4.39 | 4.39-4.39 | 343.532 | 121.15-588.68 | 36.91 | 4.39-53.68 | 0.042 |
| **IL-33, pg/ml** | 6.25 | 6.25-1,570.96 | 292.16 | 206.08-439.39 | 673.27 | 352.40-1,686.32 | 0.149 |
| **CXCL10, pg/ml** | 4384.183 | 2,533.96-8,652.31 | 4,746.06 | 2,721.07-721,819.67 | 44,791.72 | 19,511.58-70,627.60 | 0.287 |
| **CCL2, pg/ml** | 4718.108 | 4,200.07-8,513.70 | 114642.24 | 30,526.99-230,528.03 | 43,256.70 | 26,994.37-59,921.57 | 0.443 |
| **CCL3, pg/ml** | 154.56 | 2.31-794.90 | 4,402.61 | 1,635.23-6,560.42 | 1,076.49 | 459.09-3,118.44 | 0.079 |
| **CCL4, pg/ml** | 46.32 | 46.32-12,231.95 | 42,826.93 | 16,148.69-77,973.66 | 13,726.33 | 8,879.11-21,813.19 | 0.079 |
| **CCL20, pg/ml** | 232.48 | 36.24-1,210.57 | 7,473.79 | 2,828.39-42,267.15 | 2,416.59 | 1,111.36-3,683.61 | 0.172 |
| **CCL19, pg/ml** | 90.28 | 39.49-864.22 | 877.20 | 212.94-22,605.97 | 662.98 | 314.84-1,223.01 | 0.856 |
| **PDGF-AA, pg/ml** | 2,368.745 | 1,085.60-2,981.70 | 1,463.312 | 924.15-3,143.21 | 1,874.58 | 1,060.99-3,377.73 | 0.689 |
| **PDGF-AB/BB, pg/ml** | 3.36 | 3.36-117.59 | 97.00 | 63.63-152.25 | 197.58 | 80.40-255.62 | 0.360 |
| **PD-L1, pg/ml** | 1,382.90 | 32.90-2,131.72 | 1,970.98 | 1,537.35-10,610.24 | 1,476.67 | 1,312.00-2,373.00 | 0.197 |
| **CCL5, pg/ml** | 107.42 | 107.42-107.42 | 107.42 | 107.42-2,464.36 | 107.42 | 107.42-4,084.42 | 0.966 |
| **TGF-α, pg/ml** | 230,00 | 99.52-292.10 | 498.85 | 436.90-1,510.72 | 481.68 | 238.28-849.56 | 0.322 |
| **TNF-α, pg/ml** | 6.39 | 6.39-285.84 | 3,301.01 | 2,502.56-3,619.20 | 468.47 | 396.13-3,142.50 | 0.110 |
| **TRAIL, pg/ml** | 2,052.80 | 947.06-6,558.58 | 5,070.34 | 2,313.12-7,680.22 | 9,495.03 | 5,282.96-20,673.83 | 0.094 |
| **VEGF, pg/ml** | 9,040.32 | 4,989.12-30,698.41 | 13,038.17 | 7,460.30-15,696.36 | 5,732.005 | 3,445.04-9,941.60 | 0.360 |
| Notes: * Comparison between non-COVID-19 and COVID-19 ARDS groups (Mann-Whitney test) | | | | |  |  |  |

**Additional Table 5.**

|  | **Hydrocortisone** | | **Hydroxychloroquine** | | **Remdesivir** | |
| --- | --- | --- | --- | --- | --- | --- |
|  | **non-COVID-19** | **COVID-19** | **non-COVID-19** | **COVID-19** | **non-COVID-19** | **COVID-19** |
|  | **n=7** | **n=14** | **n=7** | **n=14** | **n=7** | **n=14** |
| **Number of patients that received the treatment before blood and BALF samples** | 3 (43%) | 3 (21%) | 0 | 4 (19%) | 0 | 1 (7%) |
| **Median number of days between treatment onset and samples (IQR)** | -1 [-2 ; -1] | 0 [-1 ; 0] |  | -2 [-3 ; -1] |  | -3 |

**Additional Figure 1.**

**Additional Figure 2.**
